# Characterization of GPR3 function in astrocytes in a preclinical AD mouse model

**DOI:** 10.1002/alz.095303

**Published:** 2025-01-09

**Authors:** Carlos Alberto Ayhllon Osorio, Diego Rolando Hernandez Espinosa, Yunhong Huang, Thais Rafael Guimarães, Amantha Thathiah

**Affiliations:** ^1^ University of Pittsburgh, Pittsburgh, PA USA; ^2^ APRINOIA Therapeutics, Boston, MA USA; ^3^ University of Pittsburgh Brain Institute, Pittsburgh, PA USA; ^4^ Pittsburgh Institute for Neurodegenerative Diseases, Pittsburgh, PA USA

## Abstract

**Background:**

Alzheimer’s Disease (AD) is neuropathologically characterized by the accumulation of Amyloid‐β (Aβ) plaques, neurofibrillary tangles, and neuroinflammation. GPR3 is a G protein‐coupled receptor (GPCR) that has been implicated in Aβ pathogenesis via β‐arrestin 2 (βarr2)‐mediated intracellular signaling. Genetic deletion of *Gpr3* reduces the Aβ plaque burden and cognitive decline in AD transgenic mice. However, *Gpr3* deficiency is also associated with adverse effects, e.g., increased anxiety‐ and depression‐like behavior. Recently, our lab demonstrated that selective inhibition of GPR3‐mediated βarr2 signaling maintains the beneficial physiological functions of GPR3‐mediated G protein‐signaling while reducing the Aβ plaque burden in the *App^NL‐G‐F^* AD knockin (KI) mouse model. We also determined that GPR3 is more abundantly expressed in astrocytes relative to neurons and microglia. Nevertheless, GPR3 function in astrocytes has not been investigated to date.

**Methods:**

We performed bulk RNAseq analysis of cortical brain samples from *App^NL‐G‐F^;* hemagglutinin‐tagged (HA)‐GPR3 (*AD KI*) and *App^NL‐G‐F^;* G protein‐biased HA‐GPR3 (*Biased AD KI*) mice at 6‐months of age. We are currently utilizing flow cytometry to analyze *Biased AD KI* relative to *AD KI* brains at 6‐, 12‐, and 18‐months of age (early‐, mid‐, and late‐stage pathology). We are labeling Aβ plaques *in vivo* with a fluorescent Congo‐red derivative, methoxy‐X04 (MX04). Three hours after MX04 administration, the mice are euthanized. One brain hemisphere is dissociated and analyzed by flow cytometry for ACSA2+/MX04^+^ astrocytes, GFAP+/MX04^+^activated astrocytes, and markers of proliferation (Ki67).

**Results:**

We determined that astrocytes exhibit increased hypertrophy and expression of *Gfap* in *Biased AD KI* mice. In accordance with these findings, we observe differential expression of several cytokines (e.g., *Il‐1β*, *Il‐5*, and *Chil3/5*), chemokines (e.g., *Ccl6*, *Ccl9*, and *Cxcl9*), and growth factors (e.g., *Fgf3*, *Pdgfd*, and *Vegfd*) in *Biased AD KI* mice, suggesting that GPR3 is involved in the crosstalk between astrocyte, microglia, and neurons. Preliminary FACS analysis also indicates differential expression of markers of astrocyte activation in astrocytes isolated from our *Biased AD KI* mice.

**Conclusion:**

These studies provide strong evidence for the astrocytic function of GPR3 in AD, which has therapeutic potential to reduce neuroinflammation and AD pathology and counteract neurodegeneration.

